# HSFAS mediates fibroblast proliferation, migration, trans-differentiation and apoptosis in hypertrophic scars via interacting with ADAMTS8: HSFAS regulates hypertrophic scars via inhibiting ADAMTS8

**DOI:** 10.3724/abbs.2023274

**Published:** 2023-11-24

**Authors:** Fang Ma, Honglin Liu, Tongtong Xia, Zhenghao Zhang, Shengchao Ma, Yinju Hao, Jiangyong Shen, Yideng Jiang, Nan Li

**Affiliations:** 1 School of Basic Medicine Ningxia Medical University Yinchuan 750004 China; 2 NHC Key Laboratory of Metabolic Cardiovascular Diseases Research Ningxia Medical University Yinchuan 750004 China; 3 Ningxia Key Laboratory of Vascular Injury and Repair Research Ningxia Medical University Yinchuan 750004 China; 4 General Hospital of Ningxia Medical University Yinchuan 750004 China; 5 Clinical Medical School Ningxia Medical University Yinchuan 750004 China

**Keywords:** fibroblast, HSFAS, hypertrophic scar, trans-differentiation

## Abstract

Hypertrophic scar (HS) is one of the most common sequelae of patients, especially after burns and trauma. The roles of regulatory long noncoding RNAs (lncRNAs) in mediating HS remain underexplored. Human hypertrophic scar-derived fibroblasts (HSFBs) have been shown to exert more potent promoting effects on extracellular matrix (ECM) accumulation than normal skin-derived fibroblasts (NSFBs) and are associated with enhanced HS formation. The purpose of this study is to search for lncRNAs enriched in HSFBs and investigate their roles and mechanisms. LncRNA MSTRG.59347.16 is one of the most highly expressed lncRNAs in HS detected by lncRNA-seq and qRT-PCR and named as hypertrophic scar fibroblast-associated lncRNA (HSFAS). HSFAS overexpression significantly induces fibroblast proliferation, migration, and myofibroblast trans-differentiation and inhibits apoptosis in HSFBs, while knockdown of *HSFAS* results in augmented apoptosis and attenuated proliferation, migration, and myofibroblast trans-differentiation of HSFBs. Mechanistically, HSFAS suppresses the expression of A disintegrin and metalloproteinase with thrombospondin motifs 8 (ADAMTS8). *ADAMTS8* knockdown rescues downregulated HSFAS-mediated fibroblast proliferation, migration, myofibroblast trans-differentiation and apoptosis. Thus, our findings uncover a previously unknown lncRNA-dependent regulatory pathway for fibroblast function. Targeted intervention in the HSFAS-ADAMTS8 pathway is a potential therapy for HS.

## Introduction

Skin is the first line of defense from external factors, and there are many destructive external stimuli that can lead to hypertrophic scar (HS), such as surgery, piercings, acne, tattooing, burns, lacerations, abrasions, vaccinations, and even insect bites [1]. HS formation occurs as a result of skin injury, which leads to esthetic destruction and functional impairment, resulting in physiological and psychological problems [ 2, 3]. Therefore, the study of the molecular mechanisms of HS formation is important to better understand the process and develop new therapies to treat hypertrophic scars, prevent impaired skin function, and improve skin appearance.

Dermal fibroblasts, the end effectors of HS formation, have been identified as some of the key mechanosensitive cells in the skin [4]. In response to wound stimulation, fibroblasts show promoted proliferation, migration and apoptosis inhibition [ 5, 6]. Transformation from fibroblasts to myofibroblasts is a critical procedure in the pathogenesis of scar formation, which is characterized by neo-expression of alpha smooth muscle actin-positive (α-SMA) and overproduction of extracellular matrix, particularly collagen I and collagen III [7]. However, the specific molecular mechanisms of dermal fibroblast functions remain to be completely identified.

Long noncoding ribonucleic acids (lncRNAs) are noncoding transcripts that are more than 200 nucleotides in length [8]. Currently, lncRNAs are increasingly drawing attention, and flourishing evidence has indicated that lncRNAs , such as MALAT1 [9], PFI [10], CDKN2B-AS1 [11], RMST [12], and DACH1 [13], participate in various physiological and pathological processes and play an important role in detecting ECM elasticity and modulating the differentiation of cells. H19 [14] and PAPPA-AS1 [15] are expressed in fibroblasts and enriched in HS. The molecular mechanisms involved in lncRNA functions are diverse, covering multiple steps in transcriptional to posttranscriptional processes [ 16, 17]. Therefore, exploring and targeting novel lncRNAs may provide potential alternative and effective therapies for hypertrophic scars.

In the current study, we profiled highly enriched lncRNAs in HS compared to NS and identified a previously unannotated human-specific lncRNA, HSFAS, that functions as a critical regulator of fibroblast proliferation, migration, apoptosis and myofibroblast trans-differentiation. Furthermore, we provided evidence that HSFAS interacts with and inhibits ADAMTS8 expression to regulate fibroblast proliferation, migration, apoptosis and myofibroblast trans-differentiation in HS formation. Our findings establish a novel lncRNA‐mediated fibroblast function and demonstrate its potential as a new therapeutic target for HS.

## Materials and Methods

### Clinical specimens

All clinical tissue specimens used in this study were obtained from the Affiliated Hospital of Ningxia Medical University. HS tissues and their corresponding adjacent normal tissues (NS) collected during surgery were frozen in liquid nitrogen and stored at ‒80°C until further use. NS were obtained from a standard distance (2‒3 cm) from the HS margin in resected tissues of patients. This study was approved by the Medical Ethics Committee of the Affiliated Hospital of Ningxia Medical University (No. 2018-087) and prior consents were obtained from all patients. All experiments were performed in compliance with relevant ethical regulations involving human participants.

### Cell culture and transfection

Primary fibroblasts were isolated from HS and NS as previously described [18]. The isolated cells were cultured in DMEM-F12 medium (HyClone, Shanghai, China) containing 10% fetal calf serum (Biological Industries, Beit-Haemek, Israel) and 1% penicillin‒streptomycin solution (Solarbio, Beijing, China) at 37°C in an atmosphere of 5% CO _2_. On the second day, adenovirus or siRNA was mixed with the cells using Lipofectamine 2000 (Thermo Scientific, Waltham, USA) according to the manufacturer’s instructions. The siRNA sequences of HSFAS are listed in Table 1.

**Table:** **Table 1** siRNA sequences for HSFAS and si-ADAMTS8

siRNA	Sequences (5′→3′)
si-HSFAS 1	CAGTGTAGCAGACTAT
si-HSFAS 2	GACTCCGTCTGACAAA
si-HSFAS 3	AGACACTGCTAACTGG
si-NC	GCTCCCTTCAATCCAA
si-ADAMTS8 1	GCAGCAGUGUGAGAAGUAUTT
si-ADAMTS8 2	CACCUUCUUUGUUCCUAAUTT
si-ADAMTS8 3	CCACCAAUUAUGGCUACAATT

### LncRNA sequencing

Total RNA was extracted from 5 paired HS and NS using Trizol reagent (TIANGEN, Beijing, China). LncRNA sequencing was performed by Beijing Biomarker Technologies Corporation (Beijing, China) according to the manufacturer’s standard protocols. The acquired raw data and array images were extracted with Agilent Feature Extraction software (version 11; Agilent, Santa Clara, USA). GeneSpring GX v12.0 (Agilent) was used for RNA quantification and subsequent data processing. The differentially expressed lncRNAs were identified through filtering based on fold changes (≥ 2) and *P* values ( *P* <0.05).

### Gene Ontology (GO) and KEGG pathway analysis

To better understand the biological functions of lncRNAs and mRNAs in HS and explore their potential mechanisms, we performed GO enrichment and KEGG pathway analyses on predicted target genes of differentially expressed lncRNAs (DElncRNAs). GO enrichment analysis of the differentially expressed genes (DEGs) was implemented using the clusterProfiler R packages. Enrichment analysis uses hypergeometric testing to find GO entries that are significantly enriched compared to the entire genome background. Briefly, GO analysis ( www. geneontology.org) consists of three components: biological process (BP), cellular component (CC), and molecular function (MF). KEGG is a database resource for understanding the high-level functions and utilities of biological systems, such as cells, organisms and ecosystems, from molecular level information, especially large-scale molecular datasets generated by genome sequencing and other high-throughput experimental technologies ( http://www.genome.jp/kegg/). ClusterProfiler R packages were used to find KEGG pathways that are significantly enriched compared to the entire genome background.

### Northern blot analysis

Northern blot was conducted using a DIG Northern Starter kit (Roche, Basel, Switzerland) by Sangon Biotech (Shanghai) Co. Ltd (Shanghai, China). Briefly, DIG-labelled probes were synthesized using a PCR DIG Probe Synthesis kit (Roche), and the primer sequences for probe preparation are listed in Table 2.

**Table:** **Table 2** Primer sequences for Northern blot analysis

Gene	Forward primer (5′→3′)	Reverse primer (5′→3′)
*HSFAS*	CTAGGCGAAAGAAATCGAAGTG	GCTAAGTTTGCCGAGTAAATCC

Total RNA (15 μg) was loaded onto 1% formaldehyde denatured gel electrophoresis, transferred to a Hybond nylon membrane (Amersham Biosciences, Buckinghamshire, UK), and fixed at 80°C for 2 h. The membrane was prehybridized in DIG Easy Hyb solution (Roche) at 50°C for 2 h and then hybridized with DIG-labelled probes at 50°C overnight. After being washed with 2× SSC at room temperature for 5 min and then with 0.1× SSC at 68°C for 15 min, the membrane was blocked in blocking solution for 1 h, incubated with antibody solution for 30 min, and detected using X-ray films.

### Fluorescence *in situ* hybridization (FISH) and immunofluorescence

LncRNA FISH Probe Mix (RiboBio, Guangzhou, China) was used to perform the FISH assay. Briefly, fibroblasts were fixed with 4% paraformaldehyde (Solarbio) and treated with protease K, glycine, and acetylation reagents. After prehybridization at 37°C for 30 min, fibroblasts were hybridized with Cy3-labelled HSFAS probes at 37°C overnight. Then, the fibroblasts were incubated with anti-ADAMTS8 antibody (Santa Cruz, Santa Cruz, USA) for 2 h at 37°C, and then FITC-conjugated secondary antibodies (Absin, Guangzhou, China) were used for signal visualization according to the standard protocols of immunofluorescence staining [19]. The nuclei were stained with DAPI. A confocal laser-scanning microscope (Zeiss, Oberkochen, Germany) was used to scan the images.

### RNA isolation from the nuclear and cytoplasmic fractions

The Ambion® PARIS™ system (Thermo Scientific) was used for the isolation of RNA from the nucleus and cytoplasm of fibroblasts. Cultured cells were first homogenized in ice-cold cell disruption buffer to prepare a total cell lysate. Since homogenization was performed quickly on ice and in the presence of detergent, both protein and RNA were purified directly from this lysate. For RNA isolation, a portion of the total cell lysate was immediately mixed with an equal volume of lysis/binding solution. Total RNA was then purified from the mixture using an RNA binding glass fiber filter. After three rapid washing steps, high-quality RNA was eluted in a concentrated form. Then, total RNA from the nucleus and cytoplasm was used for quantitative reverse transcription PCR (qRT-PCR) analysis.

### qRT-PCR analysis

Total RNA was extracted from human scar tissues and scar-derived fibroblasts using Trizol reagent (TIANGEN). Isolated RNA was reverse transcribed to cDNA using PrimeScript RT Master Mix (TaKaRa, Dalian, China). Quantitative PCR was performed using TB Green qPCR Master Mix (TaKaRa) on a Real-Time PCR detection system (Thermo Scientific). A total volume of 20 μL was subjected to the following PCR conditions: 95°C, 30 s for initial denaturation, 35 cycles of 95°C for 5 s and TM for 34 s. Relative expression was calculated using the 2 ^–ΔΔCt^ method and normalized to that of the housekeeping gene *GAPDH*. The sequences of primers are shown in Supplementary Table S1.

### Rapid amplification of cDNA ends (RACE)

The full-length cDNA sequence of HSFAS was obtained from fibroblast RNA using the SMARTer RACE 5′/3′ kit (TaKaRa). Nested 5′ and 3′ RACE products were obtained using a GeneRacerTM kit (Invitrogen, Carlsbad, USA). The sequences of the designed gene-specific primers for the PCR step of the RACE procedure are presented in Supplementary Table S2. PCR products were extracted using a SanPrep Column DNA Gel Extraction kit (Sangon Biotech), cloned and inserted into the pGM-T vector, and analyzed by Sanger sequencing.

### Western blot analysis

To obtain total protein, cells were lysed in lysis buffer containing protease inhibitor, phosphatase inhibitor and PMSF (Keygen Biotech, Nanjing, China). Equal amounts of protein (30 μg) were separated by 10% SDS-PAGE and then electroblotted onto PVDF membranes (Millipore, Billerica, USA). After being blocked with 5% BSA in Tris-buffered saline with 0.1% Tween 20 (TBST) for 4 h at 4°C and washed with DPBS three times, the membranes were incubated overnight at 4°C with the indicated primary antibodies. The membranes were washed again and incubated with the corresponding HRP-conjugated secondary antibody. Finally, the protein bands were visualized with the ChemiDoc™ XRS+System. Detailed information of the primary antibodies used is shown in Supplementary Table S3.

### Cell Counting Kit-8 (CCK-8) assay

Fibroblasts were inoculated into a 96-well plate at a density of 3000 cells/well. After cell culture for 24 h, 10 μL CCK8 reagent (APExBIO, Houston, USA) was added to each well. After incubation at 37°C for 2 h, the OD value was detected at 450 nm with a microplate reader (BioTek, Winooski, USA), and the data were recorded for analysis.

### 5-Ethynyl-2′-deoxyuridine (EdU) assay

Cell proliferation was determined by EdU assay using the EdU labelling/detection kit (RiboBio). A total of 200 μL of diluent A was added to each confocal dish inoculated with cells and incubated in the cell incubator for 2 h. Cells were washed with PBS, fixed with 4% paraformaldehyde for 30 min, and decolorized with 200 μL of 2 mg/mL glycine for 5 min on a shaker at room temperature. Then, 200 μL of 0.5% Triton X-100 was added to each well. After being washed once with PBS, each well was incubated with 200 μL of 1× Apollo stain in the dark for 30 min. Then, 0.5% Triton X-100 was added to each well and incubated for 10 min. Each well was stained with 200 μL of diluent F and then washed twice with PBS. After staining, cells were examined under a laser confocal microscope (Zeiss).

### Wound-healing assay

Fibroblast migration was analyzed using a wound-healing assay. Briefly, cells were cultured in 6-well plates and grown to 100% confluence. A scratch wound was created using a 1000-μL pipette tip, and the cells were treated with si-HSFAS or Ad-HSFAS based on the experimental requirements for 24 h. Images were captured under a microscope (Thermo Scientific).

### Flow cytometry

Cell apoptosis was detected by flow cytometry using a 7-amino-actinomycin (7-AAD)/propidium iodide (PI) apoptosis kit (BD, Franklin Lakes, USA) according to the manufacturer’s instructions. Briefly, fibroblasts were harvested using trypsin and washed with ice-cold PBS. Then, the fibroblasts were resuspended in 300 μL binding buffer and successively double-stained with 7-AAD (5 μL, 15 min) and PI (5 μL, 5 min) in the dark at room temperature. The fibroblasts were subjected to apoptosis analysis using an ACEA NovoCyte flow cytometry system (Agilent).

### TdT-mediated dUTP nick-end labelling (TUNEL) staining

After transfection, fibroblasts were fixed with 4% paraformaldehyde for 30 min. Then, the fibroblasts were treated with TUNEL reagent (Roche) at 37°C for 1 h. The nuclei were visualized with DAPI. After being washed with PBS, the fibroblasts were detected using a laser confocal microscope (Zeiss).

### RNA pulldown assay

*In vitro* biotin-labelled HSFAS RNA was prepared by RiboBio. Briefly, biotin-RNA was incubated with fibroblast extract (2 mg total protein) in RIP buffer with gentle rotation for 1 h at room temperature, and then the mixture was incubated with streptavidin magnetic beads (Thermo Scientific) at room temperature for another 1 h. After rigorous washing in RIP buffer with a magnetic stand, the HSFAS RNA co-precipitated protein was extracted for PAGE and immunoblotting. Silver-stained bands were cut from PAGE gels for MS analysis by liquid chromatography-MS.

### MS analysis

To identify the proteins, the enriched bands were cut out from the gel, and digested with trypsin. The prepared sample was injected into the chromatography column, where the compounds were separated based on their interactions with the stationary phase. Separated compounds were eluted from the column using a mobile phase, which carried the compounds through the column and then ionized by the mass spectrometer, typically using electrospray ionization. The ionized compounds were subsequently separated based on their mass-to-charge ratio within the mass spectrometer, allowing for the determination of their molecular weight and structure, generating a mass spectrum that provides information about the abundance of different compounds in the sample.

### RNA immunoprecipitation (RIP)

The RIP RNA-Binding Protein Immunoprecipitation kit (Millipore) was used for RIP assay, with the manufacturer’s instructions strictly followed. Cells were lysed and then incubated with magnetic beads conjugated to anti-ADAMTS8 antibody or the negative control IgG. The immunoprecipitated RNA was isolated and analyzed by qRT-PCR to assess the enrichment of target genes.

### Statistical analysis

Data were analyzed using GraphPad Prism 6. Comparisons between two groups were performed using Student’s *t* test (two-tailed), while analyses comparing multiple treatments with other groups were performed using two-way ANOVA with Tukey’s multiple comparisons test. Data are presented as the mean±SD derived from three independent experiments. *P*<0.05 was considered statistically significant.

## Results

### Altered lncRNA expression is associated with extracellular matrix (ECM) deposition and fibroblast proliferation

**Figure 1:**
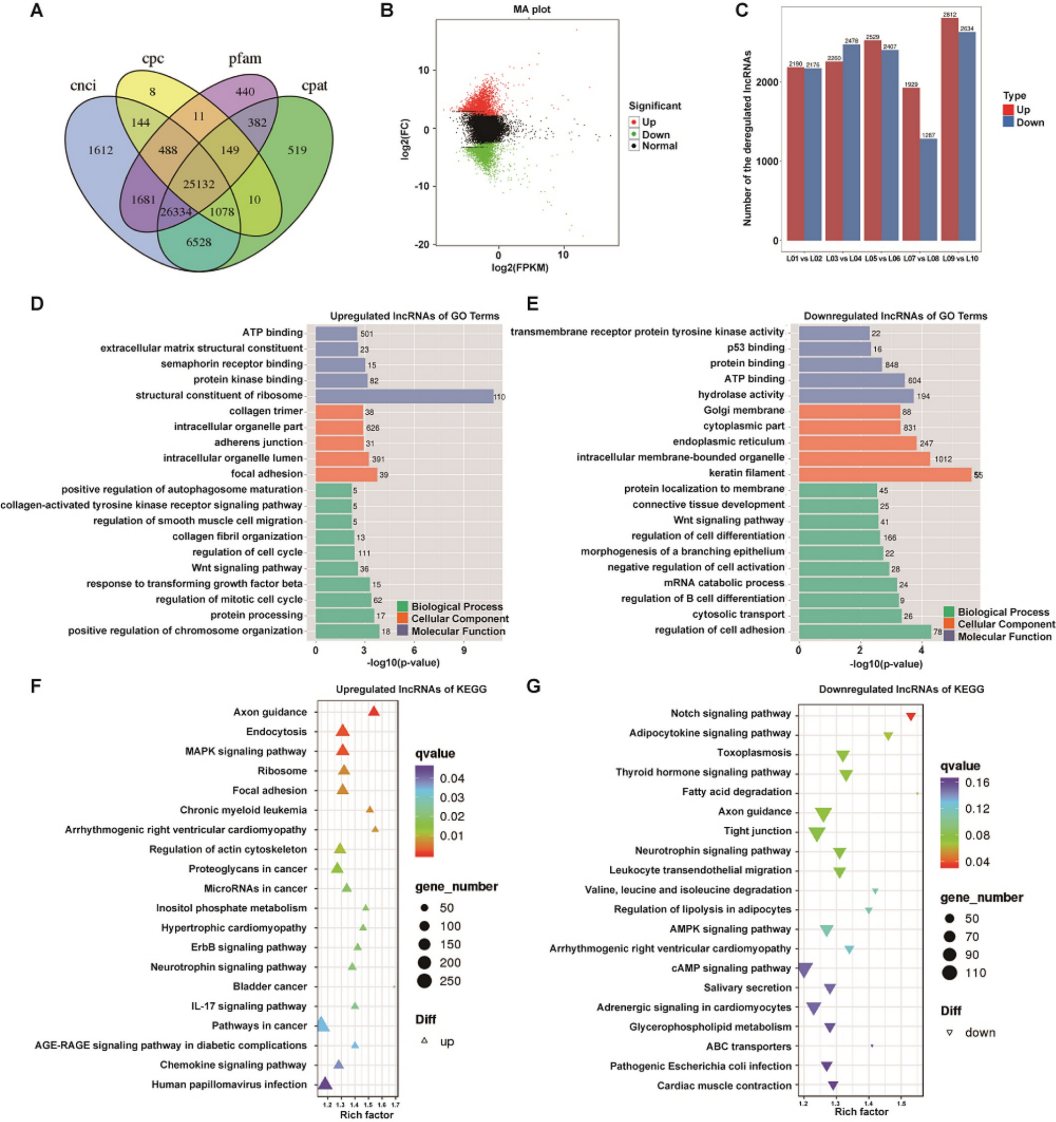
Figure 1 Profile of lncRNA expression in HS tissues and functional analysis of dysregulated lncRNAs (A) Screening of candidate long noncoding RNAs (lncRNAs) in the skin transcriptome. The Coding Potential Calculator (CPC), Coding-Non-Coding-Index (CNCI), Pfam-scan (PFAM) and Coding Potential Assessment Tool (CPAT) were used to analyze the coding potential of lncRNAs. (B) Volcano plot depicting the differential expression of lncRNAs in HS and matched adjacent NS tissues. (C) The number of up- and downregulated lncRNAs across five comparisons: L01 vs L02, L03 vs L04, L05 vs L06, L07 vs L08, and L09 vs L10. (D) Bar diagram of GO enrichment analysis of the upregulated lncRNAs. The X-axis denotes detailed annotation classes of GO ontologies by different colors, and the Y-axis denotes the percentage of lncRNAs. (E) Bar diagram of GO enrichment analysis of the downregulated lncRNAs. (F) Dots diagram of KEGG enrichment of the upregulated lncRNAs. The X-axis indicates the proportion of differentially expressed lncRNAs annotated to KEGG terms to all KEGG annotated differentially expressed lncRNAs, and the Y-axis represents the detailed classification of KEGG. The size and color of the dots represent the percentage of lncRNAs and the significance level of the adjusted P value, respectively. (G) Dots diagram of KEGG enrichment of the downregulated lncRNAs. GO, Gene Ontology; KEGG, Kyoto Encyclopedia of Genes and Genomes; BP, biological process; CC, cellular component; MF, molecular function.

To explore the roles of lncRNAs during hypertrophic scar formation, we profiled lncRNA alterations using RNA-Seq in 5 paired HS and NS tissues. As the identification of transcripts included identified and presumed lncRNA fragments, we used four tools (CPC, CNCI, pfam and phyloCSF) to remove potential coding transcripts ( Figure 1A). Finally, a total of 1739 lncRNAs (891 upregulated lncRNAs and 848 downregulated lncRNAs) were identified and subjected to further analysis ( Figure 1B). As shown in Figure 1C, there were different lncRNA expression levels across the five matched skin tissues. Then, the upregulated and downregulated lncRNAs were classified into three categories: biological process (BP), cellular component (CC), and molecular function (MF) in the Gene Ontology (GO) database. The results indicated that these upregulated lncRNAs were significantly associated with extracellular matrix (ECM) deposition and cell proliferation. We found “extracellular matrix structural constituent” and “collagen trimer” in the MF and CC parts of the GO analysis. Additionally, the enriched BP terms “collagen-activated tyrosine kinase receptor signaling pathway”, “regulation of smooth muscle cell migration”, “collagen fibril organization”, “regulation of cell cycle”, “Wnt signaling pathway” and “response to transforming growth factor beta” were all related to ECM and cell proliferation ( Figure 1D). When compared to these upregulated lncRNAs, the GO analysis of downregulated lncRNAs showed that most of them were associated with cell differentiation, such as “regulation of cell differentiation”, “morphogenesis of a branching epithelium” and “regulation of B-cell differentiation” ( Figure 1E). KEGG enrichment analysis was further implemented to identify the significantly enriched biological processes in dysregulated lncRNAs. The top 20 signaling pathways showed that many pathways of upregulated lncRNAs related to metabolism and human diseases were significantly enriched ( Figure 1F). Of them, the MAPK signaling pathway had a high enrichment, which is known to be associated with the progression of cell proliferation. Meanwhile, the top 20 signaling pathways of downregulated lncRNAs were largely likely correlated with metabolism and adipogenesis ( Figure 1G). Among them, the Notch signaling pathway had the highest enrichment. Collectively, the predicted GO and KEGG results hinted at the possible effects of lncRNAs on the pathogenesis of HS, and the upregulated lncRNAs were subject to further analysis.

### HSFAS is highly expressed in the cytoplasm and may correlate with the function of fibroblasts in HS

**Figure 2:**
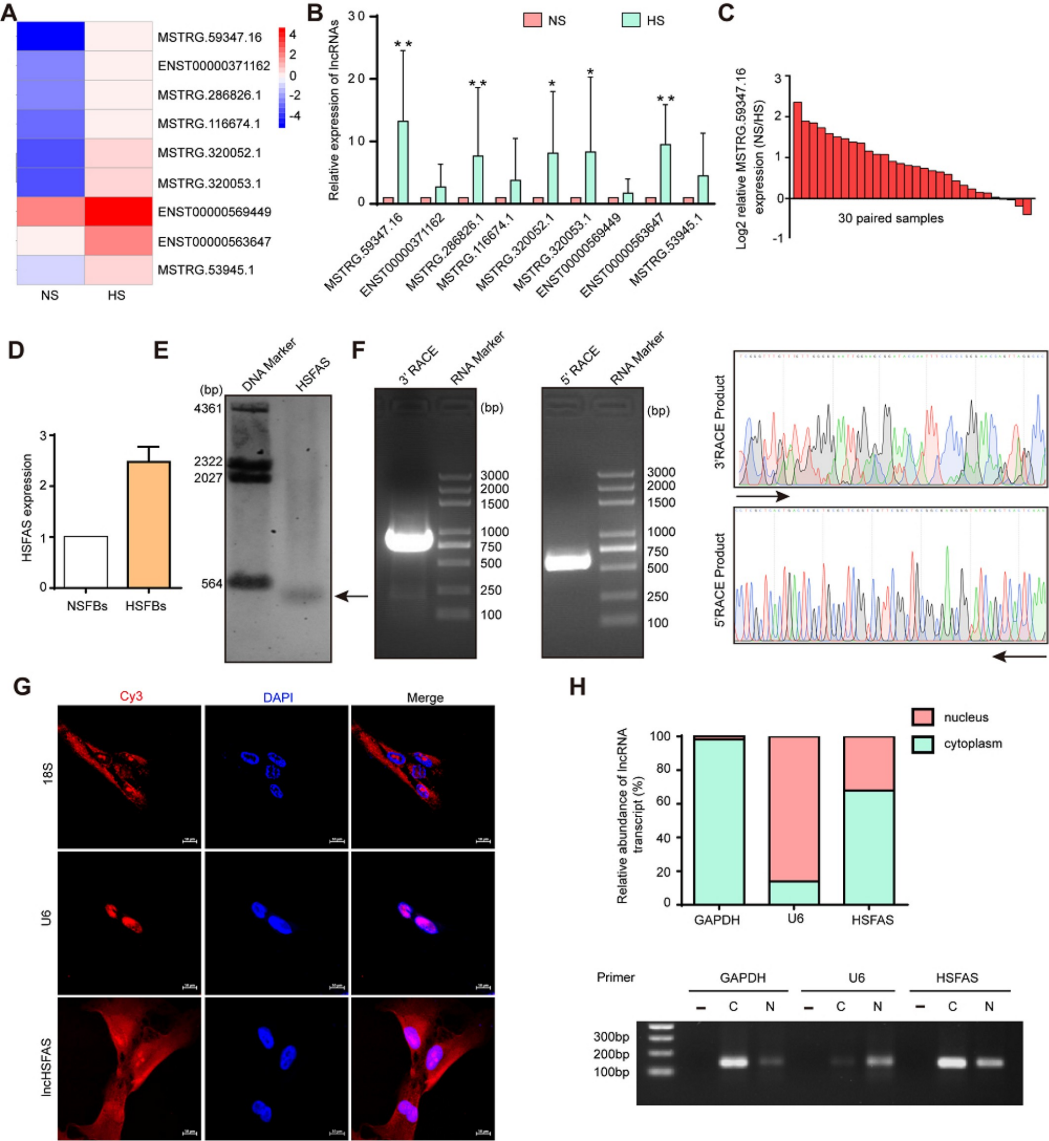
Figure 2 HSFAS is highly expressed in the cytoplasm of fibroblasts (A) A hierarchical clustering heatmap presenting the top 9 upregulated lncRNAs in HS relative to NS. (B) The expressions of 9 significantly deregulated lncRNAs, including lncRNA MSTRG.59347.16, in 30 paired NS and HS samples measured by qRT-PCR. n=30. (C) The expression of HSFAS in 30 paired NS and HS samples measured by qRT-PCR. n=30. (D) The expression of HSFAS in primary fibroblasts from 3 paired NS and HS tissues (NSFBs and HSFBs) measured by qRT-PCR. (E) Northern blot analysis of the expression of HSFAS in fibroblasts. (F) 5′-RACE and 3′-RACE assays were performed to determine the transcriptional initiation and termination sites of HSFAS. Left, representative images of the PCR products obtained by 5′-RACE and 3′-RACE. Right, the sequence of the PCR products revealed the boundary between the universal anchor primer and the HSFAS sequence. (G) FISH analysis of the subcellular distribution of lnHSFAS in fibroblasts. Scale bar: 20 μm. (H) Cytoplasmic (C) and nuclear (N) RNAs were separated and reverse transcribed to generate cDNA. PCR was conducted to detect the expression of GAPDH, U6, and HSFAS in both cytoplasmic and nuclear cDNAs. A reaction without template cDNA (‒) was conducted as a negative control. Agarose gel electrophoresis was used to visualize the PCR product. *P<0.05, **P<0.01.

Next, we selected the 9 most upregulated lncRNAs with log2FC>2 and *P*<0.01, an expression level greater than 10, and an RPKM greater than 1 in at least one group, which are shown in a hierarchical clustering heatmap ( Figure 2A). qRT-PCR analysis was performed to determine their expressions in 30 paired HS and NS samples. The results showed that 5 of them were significantly upregulated in HS tissues, of which lncRNA MSTRG.59347.16 had the highest expression level and was therefore selected for further study ( Figure 2B). Figure 2C showed the expression of lncRNA MSTRG.59347.16 in the 30 paired HS and NS samples. Additionally, we cultured and identified human primary fibroblasts from HS and NS in our previous study [18] and found that lncRNA MSTRG.59347.16 was obviously elevated in fibroblasts from hypertrophic scar tissues (HSFBs) compared to matched adjacent normal tissues (NSFBs) ( Figure 2D), suggesting that this lncRNA may play an essential role in the fibroblast function of HS. Therefore, we named it hypertrophic scar fibroblast-associated lncRNA (HSFAS).

We first confirmed HSFAS expression in fibroblasts by Northern blot analysis ( Figure 2F). Then, as a novel lncRNA, the full sequence of HSFAS needs to be determined. We characterized HSFAS by 5′ and 3′ rapid amplification of cDNA ends (RACE) experiments in fibroblasts. The alignment results indicated that the full length of HSFAS is 1679 bases ( Figure 2E and Supplementary Figure S1A). Furthermore, the 1679 bp HSFAS sequence was subjected to a battery of bioinformatics analyses. The computational algorithms CPAT, CPC2 and CNCI predicted that HSFAS has very low coding potential ( Supplementary Figure S1B). Emerging evidence has revealed that the open reading frames (ORFs) inside lncRNAs can also encode micro peptides. We assessed HSFAS for the presence of ORFs. However, there is no ORF longer than 200 bp in the sequence. Predicted peptide sequences were used to query the UniProtKB and Swiss-Port databases, but no similarities to known proteins were found ( Supplementary Figure S1C). In addition, based on the UCSC Genome Browser, HSFAS is located on chromosome 10 (q26,12) and is highly conserved among mammals ( Supplementary Figure S1D). Given that the subcellular localization of lncRNAs plays a vital role in predicting their molecular function, we evaluated the subcellular localization of HSFAS. FISH demonstrated that HSFAS was expressed in both cytoplasmic and nuclear locations but was also mainly enriched in the cytoplasm ( Figure 2G). Cytoplasmic and nuclear RNA extraction experiments followed by RT-qPCR analysis showed that approximately 70% of the HSFAS transcripts were in the cytoplasm of fibroblasts ( Figure 2H). Taken together, these results indicate that HSFAS plays a vital role in fibroblasts of hypertrophic scars.

### HSFAS promotes fibroblast proliferation, migration, and trans-differentiation to myofibroblasts and inhibits apoptosis

**Figure 3:**
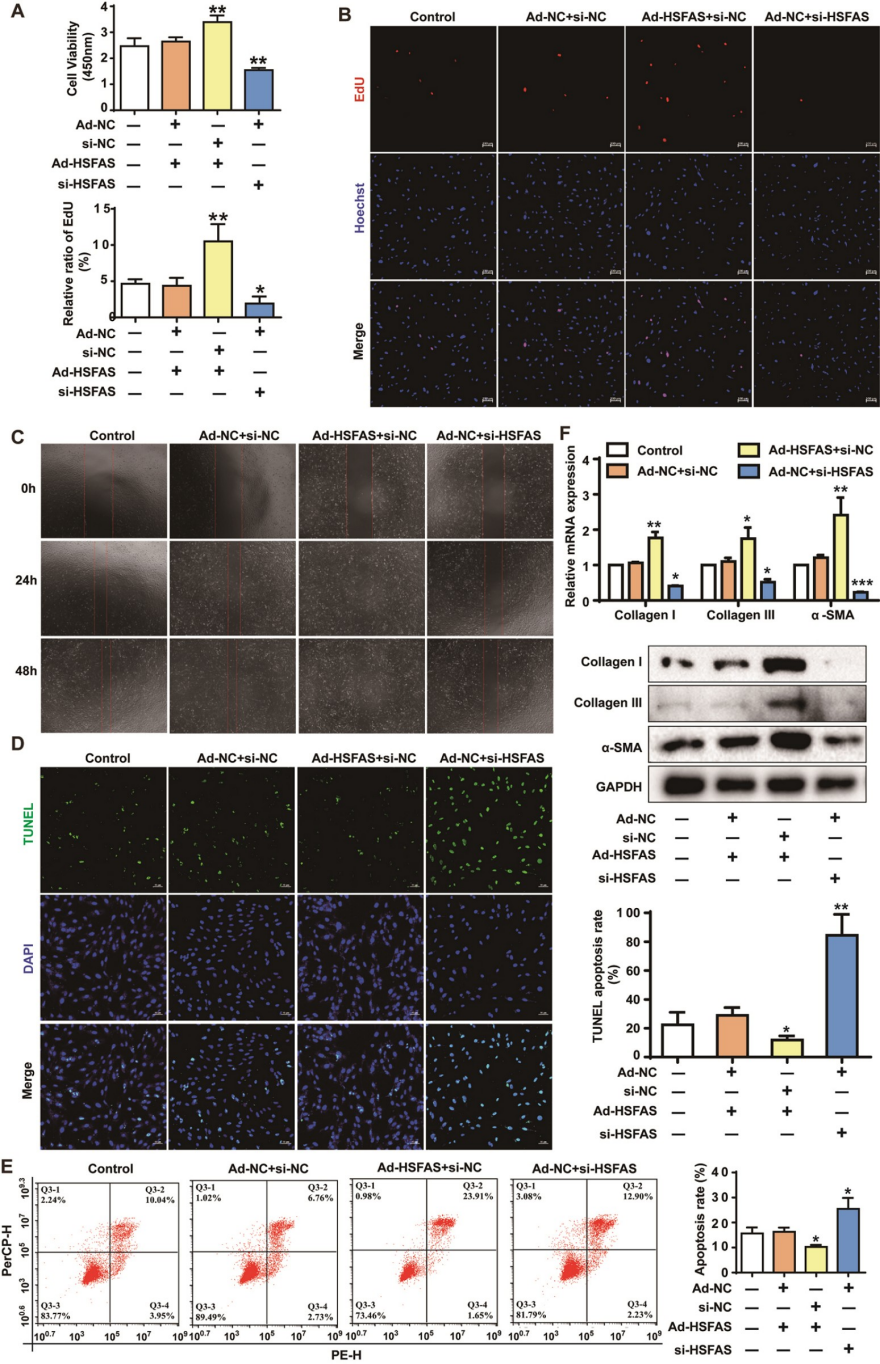
Figure 3 HSFAS promotes fibroblast proliferation and migration, trans-differentiated to myofibroblasts and inhibits apoptosis (A) Cell viability of fibroblasts detected by CCK8 assay after infection with Ad-HSFAS or transfection with si-HSFAS. (B) EdU staining assays were applied to detect the proliferation of fibroblasts infected with Ad-HSFAS or transfected with si-HSFAS. Scale bar: 100 μm. (C) Wound-healing assays were used to detect the migration of fibroblasts with HSFAS knockdown or overexpression. The cells were imaged at the same position in 6-well plates at 3 different time points (0, 24 and 48 h). (D) Apoptosis was determined by TUNEL staining following HSFAS knockdown or overexpression. Scale bar: 50 μm. (E) Apoptosis was evaluated by flow cytometry in response to HSFAS knockdown or overexpression. (F) qRT-PCR and western blot analysis of collagen I, collagen III and α-SMA expressions in fibroblasts infected with Ad-HSFAS or transfected with si-HSFAS. *P<0.05, **P<0.01, ***P<0.001.

According to the above results, siRNAs against HSFAS (si-HSFAS) and HSFAS-overexpressing adenovirus (Ad-HSFAS) were constructed to further investigate the biological function of HSFAS in HS ( Supplementary Figure S2A,B). CCK8 assays and EdU staining were used to detect the viability of fibroblasts after transfection with si-HSFAS or infection with Ad-HSFAS. As shown in Figure 3A,B, knockdown of *HSFAS* attenuated the growth of fibroblasts, while HSFAS overexpression significantly enhanced fibroblast proliferation. Wound healing assays also proved that low HSFAS level impeded the migration of fibroblasts, whereas HSFAS overexpression showed an increased tendency after fibroblasts were infected with Ad-HSFAS ( Figure 3C). Moreover, when *HSFAS* was knocked down in fibroblasts, an enhanced fluorescence intensity of TUNEL during apoptosis was captured by fluorescence microscopy ( Figure 3D). Apoptosis was also assessed by Annexin V-FITC/PI staining. As shown in Figure 3E, the percentage of cell apoptosis in fibroblasts transfected with si-HSFAS was increased. In contrast, HSFAS overexpression attenuated the percentage of cell apoptosis. These results indicated that HSFAS elevates proliferation and inhibits apoptosis of fibroblasts in HS. Furthermore, the expression levels of collagen I, collagen III and α-SMA were used to determine the effect of HSFAS on fibroblasts trans-differentiated to myofibroblasts ( Figure 3F). The mRNA and protein expression levels of collagen I, collagen III and α-SMA were significantly decreased in fibroblasts transfected with si-HSFAS, while Ad-HSFAS increased the expressions of collagen I, collagen III and α-SMA, indicating that HSFAS modulates fibroblast trans-differentiation to myofibroblasts. Taken together, these results demonstrated that HSFAS promotes fibroblast proliferation, migration, and trans-differentiation to myofibroblasts and inhibits apoptosis.

### HSFAS binds with ADAMTS8 and suppresses ADAMTS8 expression

**Figure 4:**
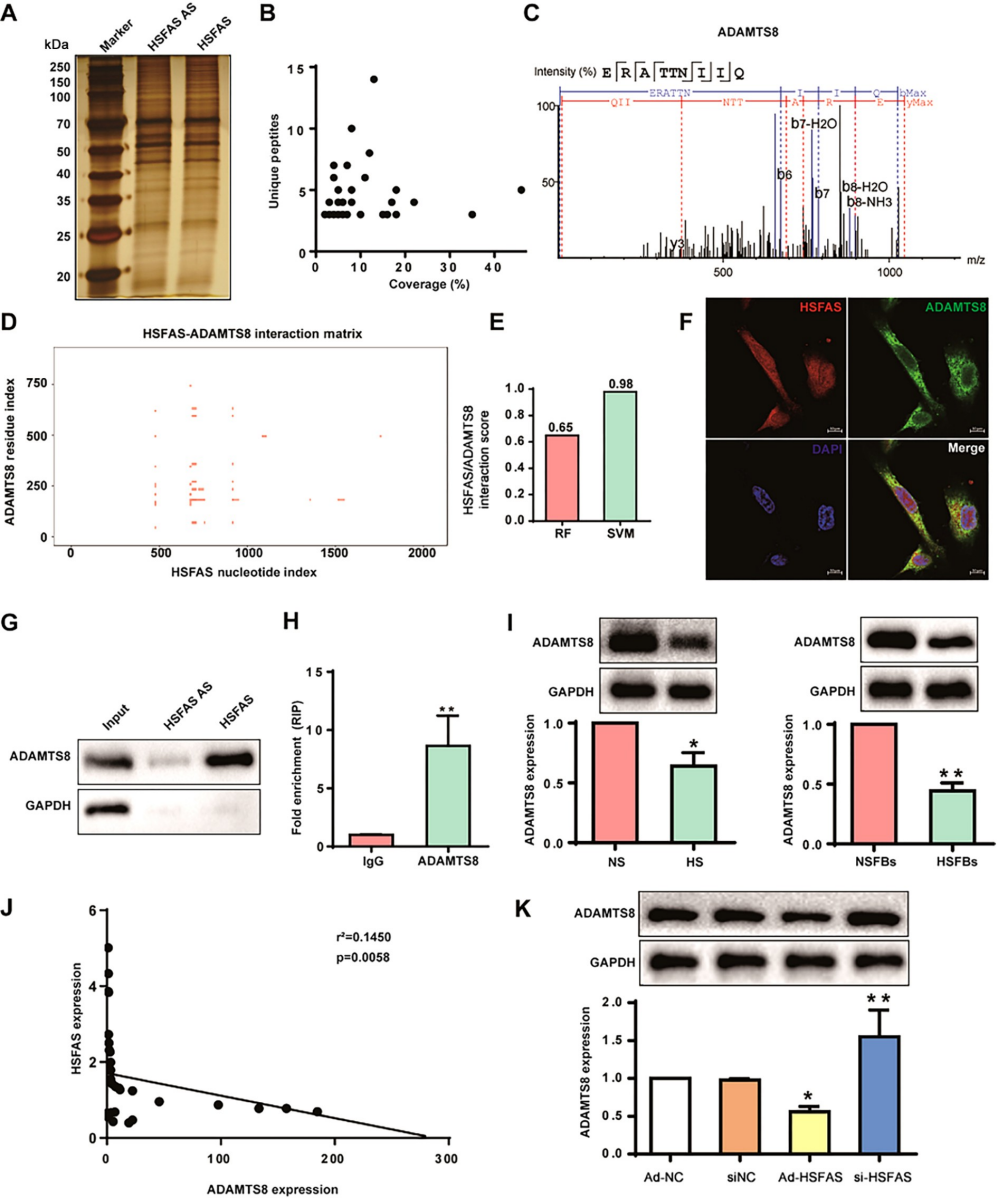
Figure 4 HSFAS binds with ADAMTS8 and suppresses ADAMTS8 expression (A) RNA pulldown assay by HSFAS and its antisense RNA followed by silver staining of protein extract from fibroblasts. AS: antisense strand of HSFAS. (B) Scatter diagram of the identified proteins by mass spectrometry (MS) analysis. Coverage (%) was plotted on the X-axis, and unique peptides were plotted on the Y-axis. (C) Unique peptides of ADAMTS8 identified by MS. (D) CatRAPID fragment module prediction of the interaction profile and matrix between HSFAS and ADAMTS8 protein (http://service.tartaglialab.com/page/catrapid_group). (E) Bioinformatics analysis of the interaction probabilities of ADAMTS8 and HSFAS binding proteins via RNA-protein interaction prediction (http://pridb.gdcb.iastate.edu/RPISeq/). Predictions with probabilities>0.5 were considered positive. RPISeq predictions are based on random forest (RF) or support vector machine (SVM). (F) Cellular localizations of HSFAS and ADAMTS8 were analyzed by combining RNA-FISH and immunofluorescence. Cell nuclei were stained with DAPI. Scale bar: 10 μm. (G) RNA pull-down assay followed by western blot analysis of the specific association of HSFAS with ADAMTS8. (H) RIP assays were carried out in fibroblasts using anti-ADAMTS8 and IgG control, followed by qRT-PCR. (I) The expression of ADAMTS8 in NS/NSFBs and HS/HSFBs. (J) Correlation between the expression of ADAMTS8 protein level and the lncHSFAS level in NS and HS tissues. The Pearson correlation coefficient and P values are listed. (K) The expression of ADAMTS8 in fibroblasts infected with Ad-HSFAS or transfected with si-HSFAS. *P<0.05, **P <0.01.

Growing evidence has indicated that many lncRNAs function through interactions with proteins [ 20, 21 ]. We performed RNA pulldown using *in vitro*-transcribed HSFAS RNA with a biotin-labelled 3′ end in fibroblasts followed by silver staining ( Figure 4A) and analyzed the protein products by mass spectrometry. With the cut-off criteria of unique peptides ≥ 3 and ‒10lgP ≥ 13, we identified 71 unique proteins that were pulled down by probes specific for HSFAS but not the negative control probe ( Figure 4B). Among all the RNA pulldown proteins, we found that HSFAS binds to A disintegrin and metalloproteinase with thrombospondin motifs 8 (ADAMTS8), which has been related to extracellular matrix organization [22], with the peptides identified by MS ( Figure 4C). Furthermore, the online databases catRAPID and RNA-protein interaction prediction (PRIseq) were used to confirm the interaction between HSFAS and ADAMTS8. catRAPID fragments, an algorithm based on individual interaction propensities of polypeptide and nucleotide sequence fragments, revealed that the 500‒1000 nucleotide positions of the HSFAS sequence may bind to the amino acid residues of the ADAMTS8 protein with high affinity ( Figure 4D). The results from PRIseq revealed overall HSFAS/ADAMTS8 interaction scores of 0.65 and 0.98, suggesting that HSFAS could interact with ADAMTS8 ( Figure 4E). Consistently, confocal microscopy demonstrated that ADAMTS8 colocalized with HSFAS in the cytoplasm of fibroblasts ( Figure 4F). RNA pulldown assays followed by western blot analysis showed that ADAMTS8 specifically bound to HSFAS ( Figure 4G). An RIP assay was further utilized to validate the specific interaction between HSFAS and ADAMTS8 ( Figure 4H). These results indicated that HSFAS could bind with ADAMTS8 in fibroblasts. We next investigated whether HSFAS has an effect on ADAMTS8 expression. First, reduced ADAMTS8 expression was observed in HS and HSFBs compared to NS and NSFBs ( Figure 4I). Pearson’s correlation analysis revealed that the expression of HSFAS was negatively correlated with ADAMTS8 expression in HS ( Figure 4J). Then, the expression of ADAMTS8 was examined in fibroblasts after *HSFAS* knockdown or overexpression, and the results showed that *HSFAS* knockdown increased ADAMTS8 expression, while HSFAS overexpression inhibited the expression of ADAMTS8 ( Figure 4K). Taken together, these results demonstrated that HSFAS binds to ADAMTS8 and suppresses ADAMTS8 expression in HS.

### HSFAS regulates fibroblast proliferation, migration, myofibroblast trans-differentiation and apoptosis by inhibiting ADAMTS8

**Figure 5:**
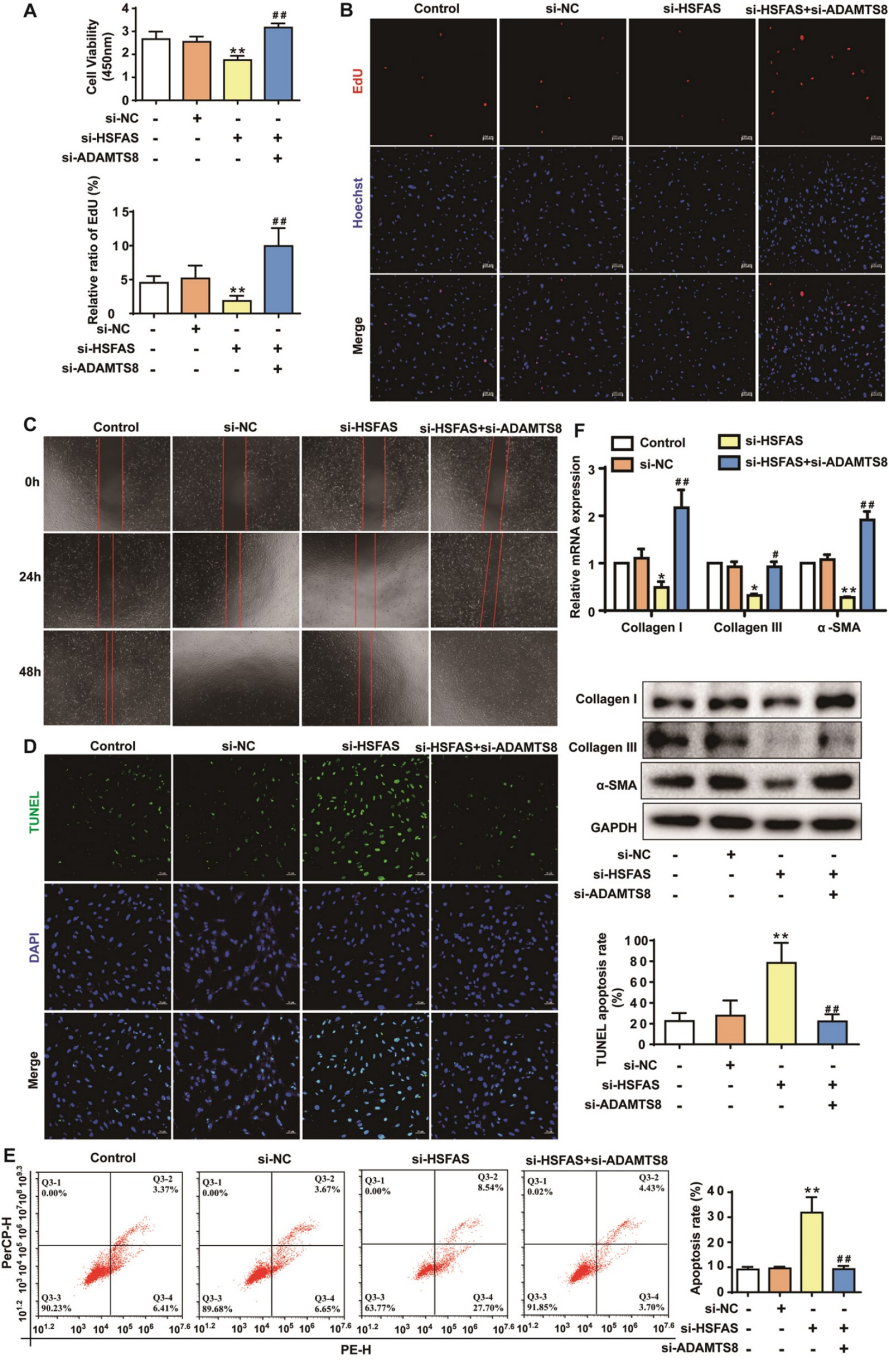
Figure 5 HSFAS modulates fibroblast proliferation, migration, myofibroblast trans-differentiation and apoptosis via ADAMTS8 (A) Cell viability of fibroblasts detected by CCK8 assay after transfection with si-HSFAS and/or si-ADAMTS8. (B) EdU staining assays were applied to detect the proliferation of fibroblasts transfected with si-HSFAS and/or si-ADAMTS8. Scale bar: 100 μm. (C) Wound-healing assays of the migration of fibroblasts with HSFAS and/or ADAMTS8 knockdown.Cells were imaged at the same position in 6-well plates at 3 different time points (0, 24 and 48 h). (D) Apoptosis was determined by TUNEL staining following HSFAS and/or ADAMTS8 knockdown. Scale bar: 50 μm. (E) Apoptosis was evaluated by flow cytometry after HSFAS and/or ADAMTS8 knockdown. (F) qRT-PCR and western blot analysis of collagen I, collagen III and α-SMA expressions in fibroblasts transfected with si-HSFAS and/or si-ADAMTS8. *P<0.05, **P<0.01 vs si-NC; #P<0.05, ##P<0.01 vs si-HSFAS.

Since HSFAS shares ADAMTS8 binding, we wondered whether ADAMTS8 is involved in the HSFAS-induced promotion of fibroblast proliferation and migration, transdifferentiated to myofibroblasts and suppression of apoptosis. The rescue experiments were carried out by co-transfection with si-HSFAS and si-ADAMTS8 ( Supplementary Figure S3). The CCK8 and EdU assays revealed that *HSFAS* knockdown inhibited fibroblast proliferation, which was reversed by silencing *ADAMTS8* ( Figure 5A,B). Increased fibroblast migration was also found in fibroblasts co-transfected with si-HSFAS and si-ADAMTS8 ( Figure 5C). Moreover, TUNEL staining and flow cytometry showed that si-HSFAS markedly increased fibroblast apoptosis, which was reversed by si-ADAMTS8 ( Figure 5D,E). Additionally, we detected the mRNA and protein expression levels of collagen I, collagen III and α-SMA in fibroblasts co-transfected with si-HSFAS and si-ADAMTS8, and the results showed that *ADAMTS8* knockdown reversed the inhibition of collagen I, collagen III and α-SMA expression induced by si-HSFAS ( Figure 5F). These results indicated that HSFAS regulates fibroblast proliferation, migration, myofibroblast trans-differentiation and apoptosis in part by regulating ADAMTS8 expression.

## Discussion

Hypertrophic scar (HS), a pathogenic form of scar formation, forms within the original wounded area developed from burns, surgeries or traumatic injuries [23]. Substantial evidence suggests that HS formation is closely related to the abnormally increased activity of fibroblasts [ 24, 25]. However, the detailed mechanism is not fully understood in fibroblasts. Thus, studies investigating the molecular mechanism of HS formation and targeted intervention are worthwhile and promising.

Recently, increasing evidence supports the importance of lncRNAs in multiple diseases, including in the regulation of hypertrophic scars [ 26‒ 28]. Here, we presented lncRNA expression profiles from 5 paired HS and matched adjacent normal tissues (NS) through RNA-Seq. The targets of these differentially expressed lncRNAs were enriched in certain signaling pathways involved in extracellular matrix (ECM) deposition and fibroblast overproliferation, such as extracellular matrix structural constituents, collagen-activated tyrosine kinase receptor signaling pathways, regulation of cell cycle, regulation of cell differentiation and MAPK signaling pathways, suggesting that these differentially expressed lncRNAs play specific roles in regulating fibroblasts in HS, which was consistent with previous reports. Moreover, a novel lncRNA, HSFAS, which was highly expressed in HS and HSFBs, was characterized for the first time. We further confirmed its expression and sequence in fibroblasts by Northern blot and RACE assays. Accumulating evidence suggests that the pathogenesis of HS is involved in the abnormally increased proliferation and migration of fibroblasts, thereby leading to apoptosis inhibition [ 29, 30]. Moreover, the trans-differentiation of fibroblasts to myofibroblasts is also a critical procedure in the pathogenesis of scar formation, which is characterized by alpha smooth muscle actin-positive (α-SMA ^+^) fibroblasts that can stimulate collagen synthesis, particularly Col I and Col III [ 31 ‒ 33]. In our study, we found that HSFAS can markedly increase the proliferation, migration, and trans-differentiation of fibroblasts to myofibroblasts, increase the levels of collagen I, collagen III and α-SMA and inhibit apoptosis.

LncRNAs have very diverse functions depending on their subcellular localization [ 34, 35]. For example, as a more cytoplasm-enriched lncRNA, TLNC1 exerts its tumorigenic function through interaction with TPR and inducing the TPR-mediated transportation of p53, finally contributing to the progression of liver cancer [36]. Another cytoplasm‐expressed lncRNA, H19, has been identified as a key hallmark of tumors, which contributes to enhancing cell growth and cell cycle by interacting with various proteins or miRNAs [37]. In this study, we found that HSFAS was located in both the nucleus and the cytoplasm of fibroblasts, mainly in the cytoplasm, implying that HSFAS mainly participates in HS formation by interacting with proteins and regulating protein functions. RNA pulldown coupled with MS analysis was performed to identify the interacting proteins of HSFAS. We also used RIP and online bioinformatics software to confirm the results. Based on MS data and online bioinformatics software, we proposed and demonstrated that ADAMTS8 interacts with HSFAS and is regulated by HSFAS. Notably, a previous study reported that ADAMTS8 is associated with cell proliferation and extracellular matrix remodeling [38]. However, whether ADAMTS8 is involved in HSFAS-regulated fibroblast functions is unknown. In this study, *ADAMTS8* silencing elevated the negative effects of *HSFAS* knockdown on the proliferation, migration and differentiation of fibroblasts to myofibroblasts and attenuated fibroblast apoptosis. Our study provides potential therapeutic targets for HS by revealing a novel signaling axis of ADAMTS8 that is regulated by HSFAS.

In summary, we provide the first lncRNA landscape of HS and NS and identify a novel lncRNA, HSFAS that is able to promote fibroblast proliferation, migration and trans-differentiation to myofibroblasts and attenuate apoptosis. Mechanistically, we demonstrate that HSFAS interacts with HSFAS to reduce ADAMTS8 expression, finally contributing to the increased functions of fibroblasts, suggesting that HSFAS and ADAMTS8 may serve as therapeutic and prognostic biomarkers for HS.

## Supporting information

23464Supplementary_Tables

23464Supplementary_Figures

## Supplementary Data

Supplementary data is available at *Acta Biochimica et Biophysica Sinica* online.
