# Supplementary material for: HSFAS mediates fibroblast proliferation, migration, trans-differentiation and apoptosis in hypertrophic scars via interacting with ADAMTS8: HSFAS regulates hypertrophic scars via inhibiting ADAMTS8
Source: Acta Biochim Biophys Sin (Shanghai). 2023 Nov 24;56(3):440–51. doi: 10.3724/abbs.2023274 (PMC10984868; doi:10.3724/abbs.2023274)
Supplement: 23464Supplementary_Tables [file 23464Supplementary_Tables.docx]

**Supplementary Table S1.** **Sequences of primers used for qRT-PCR**

| Gene | Forward primer (5′→3′) | Reverse primer (5′→3′) |
| --- | --- | --- |
| ENST00000569449 | GCTACAGTTGGCTAGTGGGTCTTC | AGCTCTGGCAGTACATGTGTCTTG |
| ENST00000563647 | TACAGTTCACGGTCCTCGGC | AAGCGCTGCATTCCACAAGT |
| ENST00000371162 | TAGGACTTCGCCGATCTCACAGG | TCACCGCAAATCCCATCCCATTG |
| MSTRG.320052.1 | TGTGAAGGCACTGCTCCATTGTC | AAAGTCGCCATCCTAAACGCTTCC |
| MSTRG.286826.1 | CAGTGTATCCCTGTTGCCTGTCTC | AGGAACTCTGGAGTGCTAGTGTGG |
| MSTRG.116674.1 | TGTCGTCTGGGCGGAGGTTG | GAGATGGAGTCTTGCTCTGTTGCC |
| MSTRG.320053.1 | TCACCTCTGCCTTCCAGTAAGTCC | AGTCCTCACCCAAAGCAAGAAACC |
| MSTRG.59347.16 | GGTGTCCTTGCCGTTTCCTTCTG | GGAGGTGGAGGTGGAGGTTACAG |
| MSTRG.53945.1 | TGGCTGCTAAGTGGGGAAGGAG | GCAAAGGCACAAACACAGGACATG |
| GAPDH | ATTCCACCCATGGCAAATTCC | GACTCCACGACGTACTCAGC |
| Collagen I | GAGGGCCAAGACGAAGACATC | CAGATCACGTCATCGCACAAC |
| collagen III | GGAGCTGGCTACTTCTCGC | GGGAACATCCTCCTTCAACAG |
| α-SMA | CTGCTGAGCGTGAGATTGTC | CTCAAGGGAGGATGAGGATG |

**Supplementary Table S2.** **Gene-specific primers for 5**′ **and 3**′**-rapid amplification of cDNA ends (RACE) analysis**

|  | rHSFAS-R1 (5′→3′) | rHSFAS-R2 (5′→3′) |
| --- | --- | --- |
| 5’-RACE | GAGGCTACTAGTTCTATTTATGGTCAGCT | GTGGTGTGTGAGATGAGCATGGAATGGTCTT |
| 3’-RACE | GCTCATCTCACACACCACAAAGGAAGCTGA | CACTGCTAACTGGATCAATGAACTCTTTATC |

**Supplementary Table S3. Detailed information of primary antibodies**

| Protein | Host | Dilution | Catalog number | Company |
| --- | --- | --- | --- | --- |
| Collagen I | Mouse | 1:3000 | Ab6308 | Abcom |
| collagen III | Rabbit | 1:5000 | Ab7778 | Abcom |
| α-SMA | Mouse | 1:5000 | ABM0052 | Abbkine |
| ADAMTS8 | Mouse | 1:1000 | sc-514717 | Santa Cruz |
| GAPDH | Rabbit | 1:10000 | Bs-2188R | Bioss |
