# Supplementary material for: HSFAS mediates fibroblast proliferation, migration, trans-differentiation and apoptosis in hypertrophic scars via interacting with ADAMTS8: HSFAS regulates hypertrophic scars via inhibiting ADAMTS8
Source: Acta Biochim Biophys Sin (Shanghai). 2023 Nov 24;56(3):440–51. doi: 10.3724/abbs.2023274 (PMC10984868; doi:10.3724/abbs.2023274)
Supplement: 23464Supplementary_Figures [file 23464Supplementary_Figures.docx]

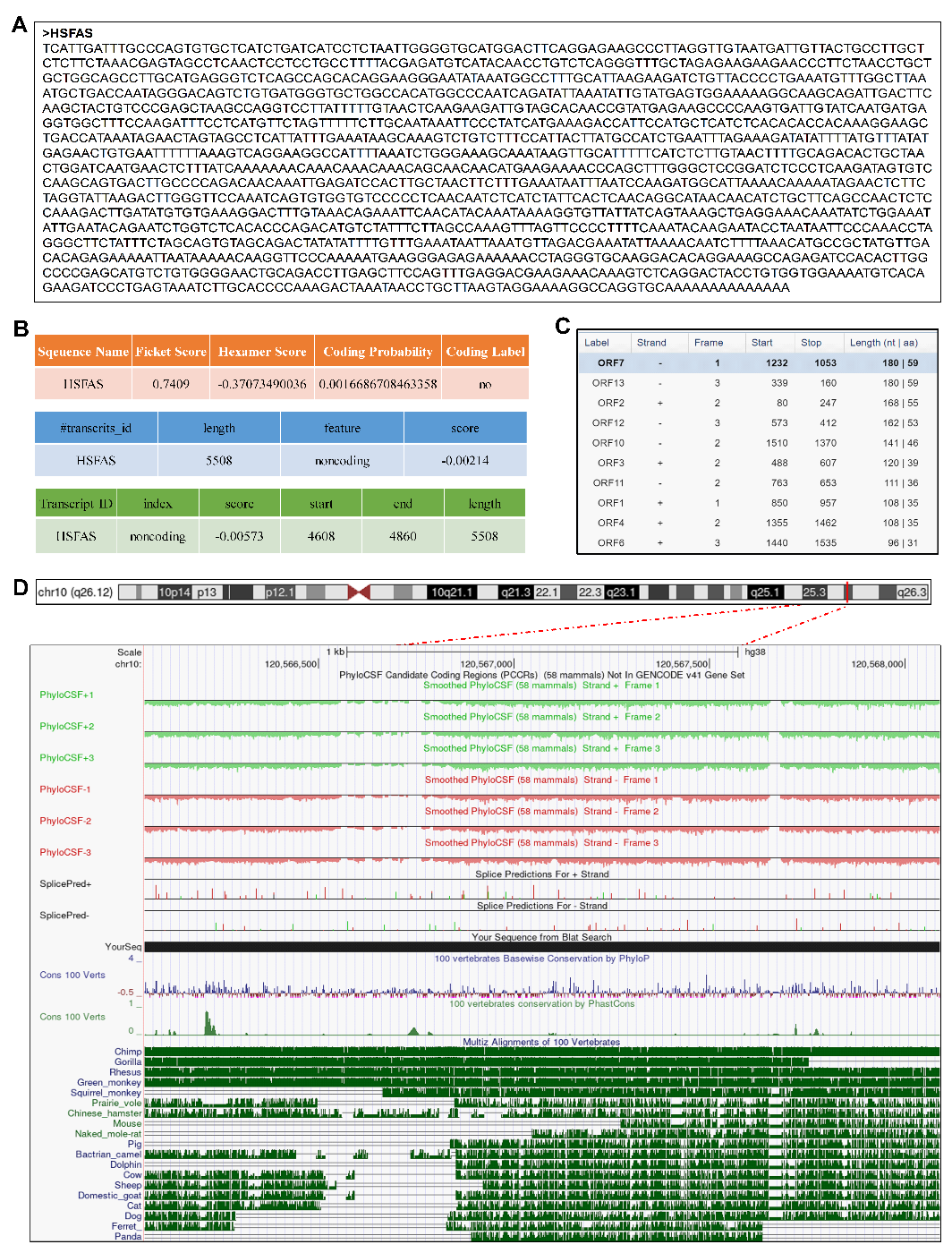


**Supplementary Figure S1. Characterization of the HSFAS** (A) The full-length RNA sequence of this transcript. (B) The coding potential of this transcript was predicted using three computational approaches (CPC, coding potential calculator; CNCI, coding-non-coding index; CPAT, coding potential assessment tool). (C) ORF finder was used to analyze the protein-coding ability of *HSFAS*. (D) Genomic location and conservation of *HSFAS* are shown on the UCSC genome browser version hg19.


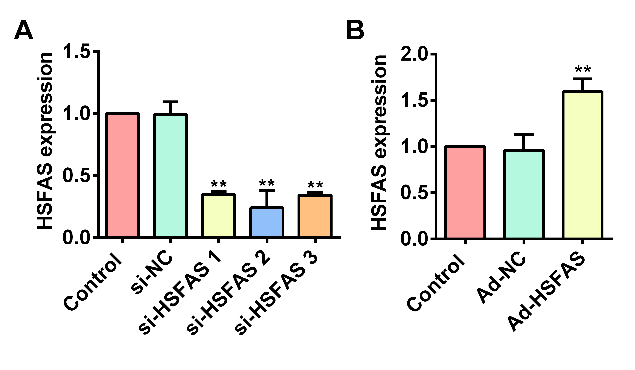


**Supplementary Figure S2. The expression of HSFAS in fibroblasts**  (A) The expression of HSFAS in fibroblasts transfected with siRNAs against HSFAS (si-HSFAS 1, si-HSFAS 2, and si-HSFAS 3) or negative control (si-NC). (B) The expression of HSFAS in fibroblasts infected with HSFAS-overexpressing adenovirus (Ad-HSFAS) or negative control (Ad-NC). ***P*<0.01.


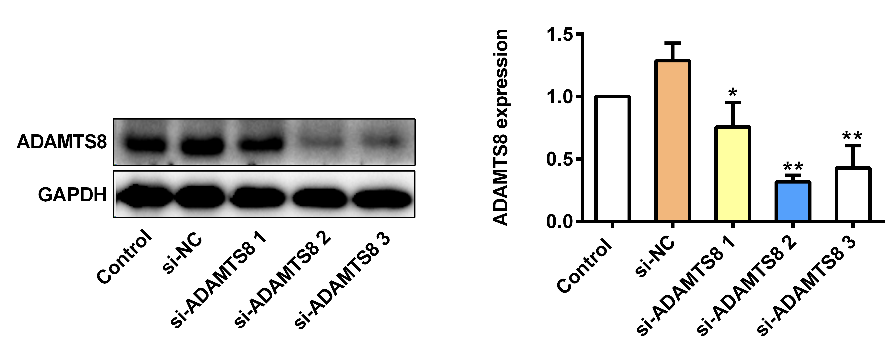


**Supplementary Figure S3. The expression of ADAMTS8 in fibroblasts transfected with siRNAs against ADAMTS8 (si-ADAMTS8 1, si-ADAMTS8 2, and si-ADAMTS8 3) or negative control (si-NC)**  **P*<0.05, ***P*<0.01.
